# Supplementary material for: Once yearly cell-based therapy for sustained and dose tunable delivery of monoclonal antibodies
Source: bioRxiv. 2026 May 21:2026.05.19.726224. Preprint. [Version 1] doi: 10.64898/2026.05.19.726224 (PMC13228534; doi:10.64898/2026.05.19.726224)
Supplement: Supplement 1 [file media-1.pdf]

Supplementary Information

SUPPLEMENTARY INFORMATION **1**

SUPPLEMENTARY TABLES 1

Table 1. Source and catalog numbers of materials, tools, and equipment used throughout this manuscript. 1

Table 2. Top 20 proteins across secretome sample groups and their function not including mAbs 3

Table 3. Reported Clearance Rates and Half-Lives for mAbs of Interest 4

SUPPLEMENTARY RESULTS 5

Supplementary cell engineering figures *5*

Supplementary Figure 1. Plasmid maps for mAb expression. 5

Supplementary capsule optimization and in vivo figures *6*

Supplementary Figure 2: Encapsulated cell quality control. 6

Supplementary Figure 3. Immunofluorescence images of unmodified SLG20 and Z2A19 modified capsules. 6

scRNA-Seq supplementary figures *7*

Supplementary Figure 4. UMAP of cluster-specific genes for myeloid cells, B cells, and T cells. 7

Supplementary Figure 5. Anti-inflammatory macrophage markers. 8

Supplementary Figure 6. Supplementary Figure 6: Resident macrophage markers. 8

Supplementary Figure 7: Inflammatory macrophage markers. 9

Supplementary Figure 8: Follicular B Cell markers 9

Supplementary Figure 10. CD4 T Cell markers. 11

Proteomics & additional mAb supplementary figures *11*

Supplementary Figure 11: Secretomics additional data 11

Supplementary Figure 12. Differences in mAb titer and pharmacokinetics in immunocompetent vs immunocompromised mice. 12

Supplementary H&E Images *13*

Supplementary Figure 13. Extended H&E images. 14

SUPPLEMENTARY METHODS 14

Small molecule synthesis *14*

NMR and elemental Analysis of Top Three Molecules *19*

Device Manufacturing *20*

Supplementary Figure 14. Lattice device fabrication and post-print processing method. 20

Supplementary Tables

Table 1. *Source and catalog numbers of materials, tools, and equipment used throughout this manuscript.*

Reagent/Resource	Source/Company	Catalog Number	Additional Information
ARPE-19 cells	ATCC	CRL-2302	Human retinal pigment epithelial cells
DMEM/F-12 medium	Cytiva	SH30023.01	1:1 formulation
Fetal bovine serum (FBS)	VWR	76236-336	10% in culture medium
Antibiotic-antimycotic	Fisher	15-240-062	1% in culture medium
Trypsin-EDTA (0.25%)	Cytiva Hyclone	SH30042.02	For passaging
Opti-MEM medium	ThermoFisher	11058021	Serum-free
Lipofectamine 3000	ThermoFisher	L3000015	Transfection reagent
Puromycin	Gibco	A1113803	2.5 µg/mL for selection

alamarBlue HS	ThermoFisher	A50101	10% solution for viability
SLG20 alginate	Sigma	42000001	Unmodified alginate
SLG100 alginate	Sigma	4202101	Unmodified alginate
Coaxial needle	Ramé-Hart	100-10-COAXIAL-2218	For core–shell capsule fabrication
Syringe pump	Harvard Apparatus	Pump 11 Pico Plus	For electrospraying capsules
$BaCl_2$ crosslinking bath chemicals	Gibco Life Technologies	—	20 mM $BaCl_2$, 250 mM d-Mannitol, 25 mM HEPES, 0.01% Tween-20
Human IgG ELISA kit	Abcam	ab195215	For quantifying antibody
Adalimumab ELISA kit	Abcam	ab237641	Antigen-specific
Ipilimumab ELISA kit	Abcam	ab237653	Antigen-specific
Pembrolizumab ELISA kit	Abcam	ab237652	Antigen-specific
COL1A1 ELISA kit	Abcam	ab210579	Pro-Collagen I alpha 1 assay
MycoProbe Mycoplasma Detection Kit	R&D Systems	CUL001B	For regular myco-plasma testing of cells in culture
3D printer (Form 4B)	Formlabs	—	Stereolithography printer
BioMed Clear resin	Formlabs	RS-C2-BMCL-01	For 3D-printed macrodevice lattice
Live/Dead Viability Kit	Invitrogen	L3224	For cell viability staining
Alginate lyase	Millipore Sigma	A1603	For hydrogel dissolution
Countess III Automated Cell Counter	ThermoFisher	AMQAX2000	For viability counts
C57BL/6J mice	Charles River	027	Immunocompetent strain
BCD mice	JAX	002288	B6.129S2-Ighmtm1Cgn/J
RAG2 mice	JAX	008449	B6.Cg-Rag2tm1.1Cgn/J
NSG mice	JAX	005557	NOD.Cg-Prkdcscid Il2rgtm1Wjl/SzJ
EthiqaXR	Fidelis	—	Buprenorphine extended-release analgesic, 1.3 mg/mL
Heating pad	Adroit	HTP-1500	For surgical warming
Anti-α-SMA-eFluor 660	Invitrogen	50-9760-82	For immunofluorescence
Anti-CD68 CoraLite 594	Proteintech	CL59425747100UL	For immunofluorescence
NucBlue (DAPI)	Invitrogen	R37606	Nuclear counterstain
Anti-CD45 Alexa Fluor 488	Abcam	ab256254	Flow cytometry marker
NucBlue (DAPI)	Invitrogen	R37606	Nuclear counterstain
Miltenyi adipose dissociation kit	Miltenyi	130-105-808	For single-cell prep
S-Trap micro column	ProtiFi	CO2-micro-80	For proteomics digestion
Pierce peptide assay	Thermo Scientific	23275	For proteomics peptide quantification
Orbitrap Eclipse MS	Thermo Scientific	—	Mass spectrometer
Vanquish Neo UHPLC system	Thermo Fisher	—	Proteomics LC system
10x Genomics Chromium	10x Genomics	—	Single-cell capture
Seurat	Open source	—	Bioinformatics software (R package)
EVOS fluorescence microscope	Invitrogen	—	For cell imaging
Nikon A1-R confocal microscope	Nikon	—	For immunofluorescence
F.Sight single-cell dispenser	Cytena	—	For monoclonal isolation

Table 2. *Top 20 proteins across secretome sample groups and their function not including mAbs*

Gene ID	UniProt ID	UniProt Function Summary
CTSD	P07339	Acid protease active in intracellular protein breakdown. Plays a role in APP processing following cleavage and activation by ADAM30 which leads to APP degradation
CST3	P01034	Cysteine protease inhibitor; regulates cathepsin activity
GSN	P06396	Actin filament severing/capping protein; regulates cytoskeleton dynamics
SERPINF1	P36955	Neurotrophic protein; induces extensive neuronal differentiation in retinoblastoma cells. Potent inhibitor of angiogenesis.it exhibits no serine protease inhibitory activity.
RARRES2	Q99960	A component of desmosome cell-cell junctions which are required for positive regulation of cellular adhesion
B2M	P61769	Component of the class I major histocompatibility complex (MHC). Involved in the presentation of peptide antigens to the immune system.
SPARC	P09486	Appears to regulate cell growth through interactions with the extracellular matrix and cytokines. Binds calcium, copper, collagen, albumin, thrombospondin, PDGF and cell membranes
EFEMP1	Q12805	Binds EGFR, the EGF receptor, inducing EGFR autophosphorylation and the activation of downstream signaling pathways. May play a role in cell adhesion and migration.
PPIA	P62937	Catalyzes the cis-trans isomerization of proline imidic peptide bonds in oligopeptides. Activates endothelial cells (ECs) in a pro-inflammatory manner
IGFBP7	Q16270	Binds IGF1 and IGF2 with a relatively low affinity. Stimulates prostacyclin (PGI2) production. Stimulates cell adhesion. Acts as a ligand for CD93 to play a role in angiogenesis
LGALS1	P17931	β-galactoside-binding lectin; mediates cell–cell and cell–matrix adhesion
ECM1	Q16610	ECM structural/regulatory protein; Stimulates the proliferation of endothelial cells and promotes angiogenesis. Inhibits MMP9 proteolytic activity.
NPC2	P61916	Lysosomal cholesterol transporter; essential for lipid metabolism
TIMP1	P01033	Inhibits matrix metalloproteinases; regulates ECM turnover
CLU	P10909	Molecular chaperone; involved in apoptosis, lipid transport, and complement regulation
PSAP	P07602	Precursor of saposins; essential for sphingolipid degradation in lysosomes
IGFBP6	P24592	Binds IGFs, preferentially IGF-II; inhibits growth, Activates the MAPK signaling pathway and induces cell migration
PTGDS	P41222	Prostaglandin D synthase; produces PGD_2 (modulates sleep, inflammation)
DKK3	Q9UBP4	Wnt signaling inhibitor
TGFBI	Q15582	ECM adhesion molecule induced by TGF-β; influences cell adhesion
FN1	P02751	Fibronectin; cell adhesion, migration, and wound healing
CHI3L1	P36222	Chitinase-3-like protein 1; tissue remodeling, inflammation
C3	P01024	Complement component 3; activates complement system

TIMP2	P16035	Metalloproteinase inhibitor; ECM homeostasis
CALR	P27797	Calreticulin; Calcium-binding chaperone that promotes folding, oligomeric assembly and quality control in the endoplasmic reticulum (ER) via the calreticulin/calnexin cycle.
CP	P00450	Ceruloplasmin; ferroxidase activity, iron homeostasis
SOD3	P08294	Extracellular superoxide dismutase; ROS regulation
MMP2	P08253	Matrix metalloproteinase-2; ECM remodeling, angiogenesis

Table 3. *Reported Clearance Rates and Half-Lives for mAbs of Interest*

mAb	CL	Source
3BNC117	390 mL/day	Approximated based on reported half-life in HIV+ patients (9 days) by Caskey et al. [1] and a blood volume of 5000 mL.
PGT121	250 mL/day	Approximated based on reported-half-life for HIV+ patients on ART (14 days) reported by Stephenson et al. [2] and a blood volume of 5000 mL.
Ipilimumab	400 mL/day	Ipilimumab FDA label
Pembrolizumab	200 mL/day	Pembrolizumab FDA label
Rituximab	340 mL/day	Rituximab FDA label
Adalimumab	290 mL/day	Adalimumab FDA label.

Supplementary Results

Supplementary cell engineering figures

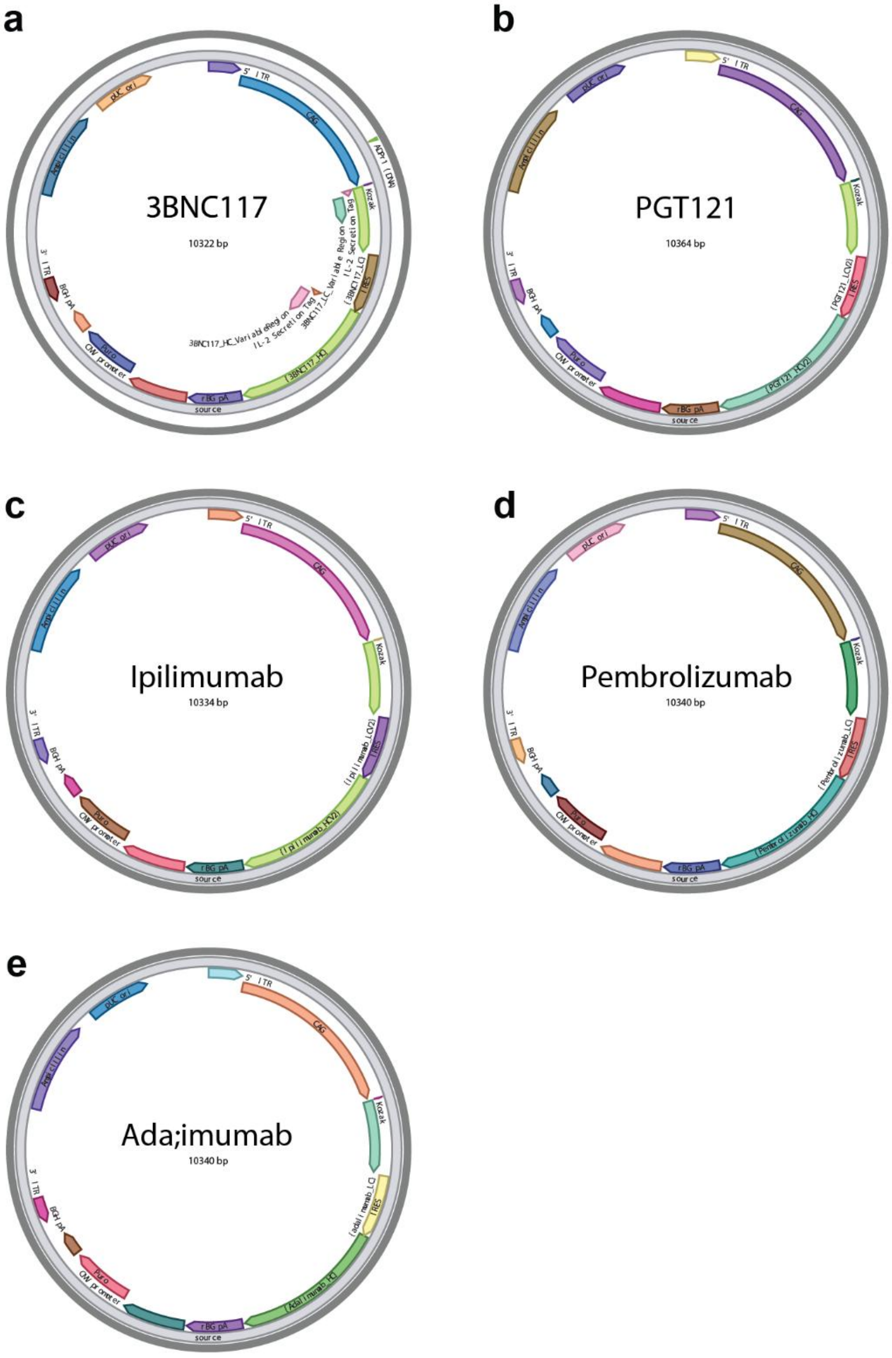

Supplementary Figure 1. Plasmid maps for mAb expression. **a-e**, Genetic constructs used to engineer cells to produce mAbs of interest 3BNC117, PGT121, Ipilimumab, Pembrolizumab, Adalimumab respectively.

Supplementary capsule optimization and in vivo figures

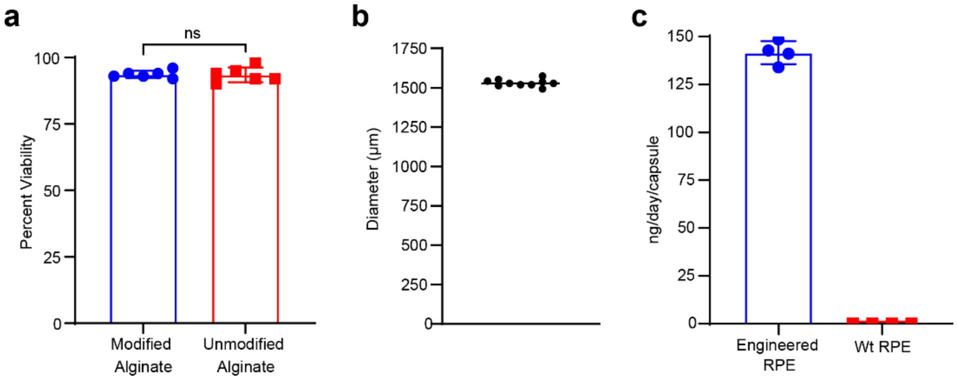

Supplementary Figure 2: Encapsulated cell quality control. a, Cell viability between modified and unmodified Alginate as determined by trypan blue staining (n=6). **b,** Capsule diameter distribution (n=10). **c,** IgG secretion rate from capsules engineered cells vs naïve ARPE-19s (n=4).

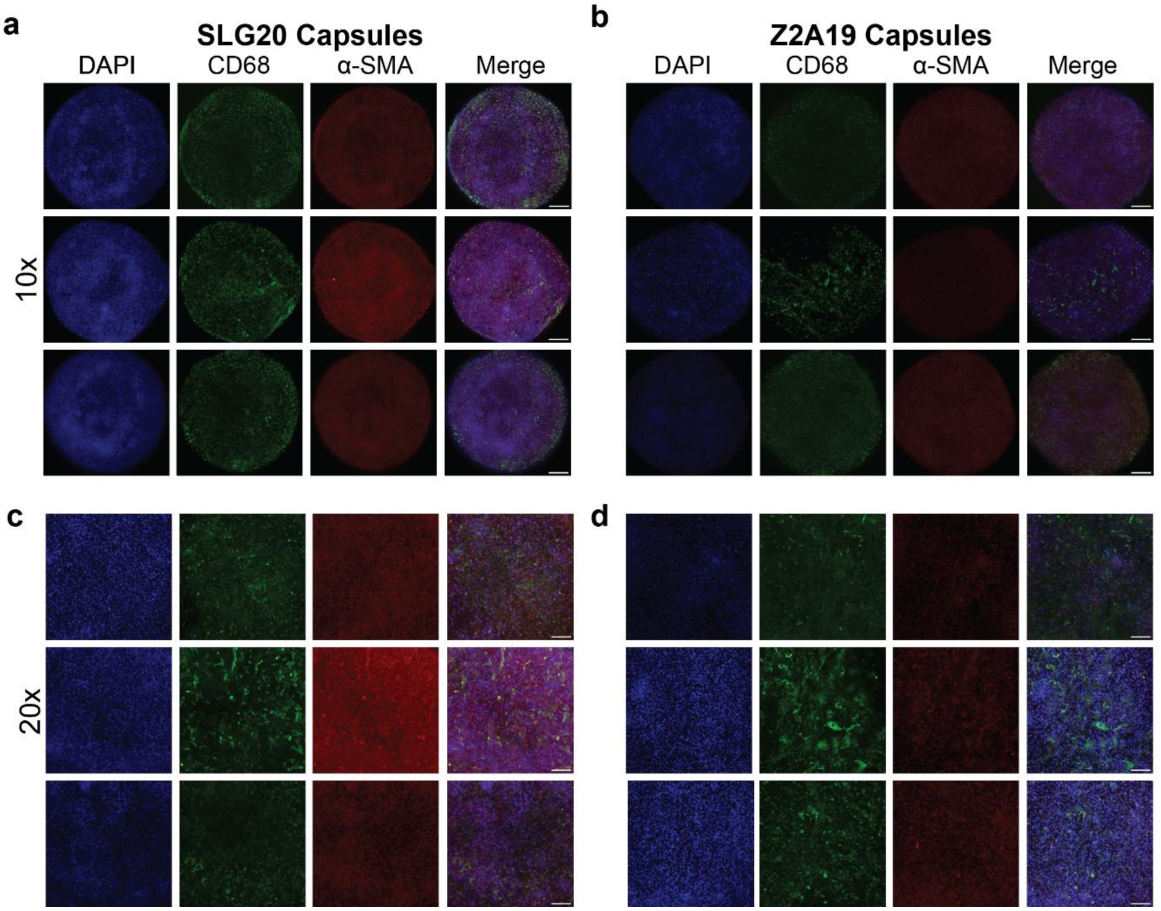

Supplementary Figure 3. Immunofluorescence images of unmodified SLG20 and Z2A19 modified capsules. Capsules were explanted from C57BL/6 mice (n=3) for imaging after 2-weeks and stained with DAPI nuclear stain, anti-CD68 to visualize macrophages, and anti-a-SMA to visualize myofibroblasts. **a-b**, show macroscale images of stitched and z-stacked capsules using a 10x objective. **c-d**, show z-stacked images of capsules obtained with a 20x objective. Scale bar for 10x images = 200 µm. Scale bar for 20x images = 100 µm.

scRNA-Seq supplementary figures

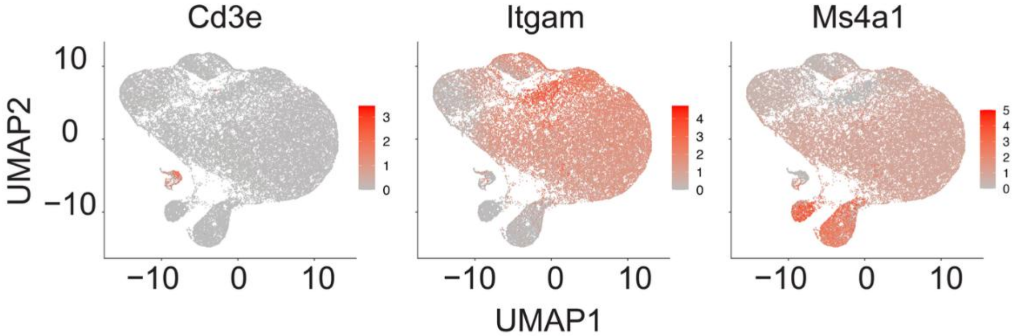

Supplementary Figure 4. UMAP of cluster-specific genes for myeloid cells, B cells, and T cells. Integrated clusters across three conditions: sham mice, mice implanted with Z2A19 material with cells, and mice implanted with SLG20 material with cells. Genes Cd3e, Itgam, and Ms4a1 are plotted for specific types of cell types (T cells, myeloid cells, and B cells respectively). Red dots indicate cells with higher gene expression while gray dots represent cells with lower gene expression.

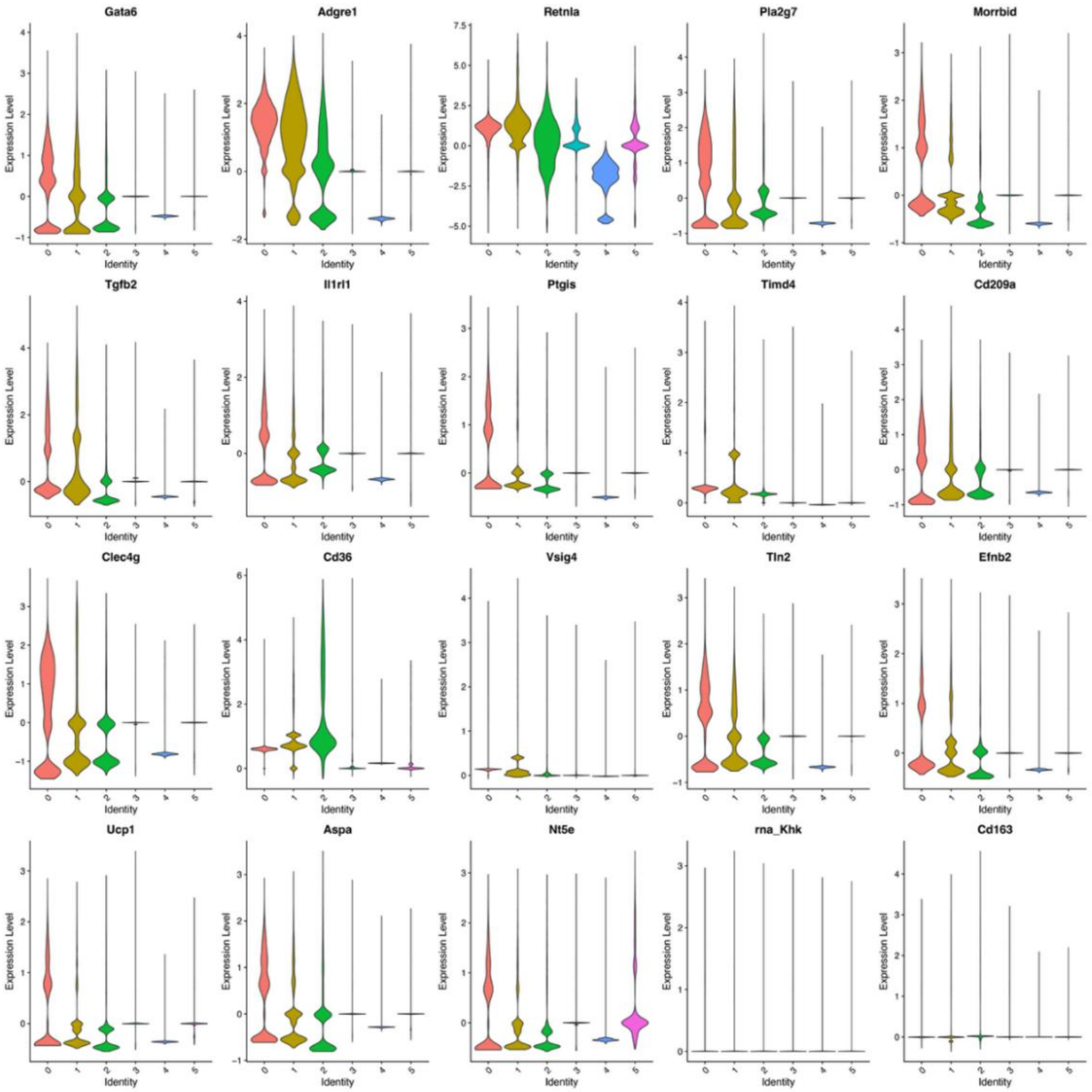

Supplementary Figure 5. Anti-inflammatory macrophage markers. Violin plot of genes associated with anti-inflammatory macrophages. Numbers on the x-axis are the cluster number.

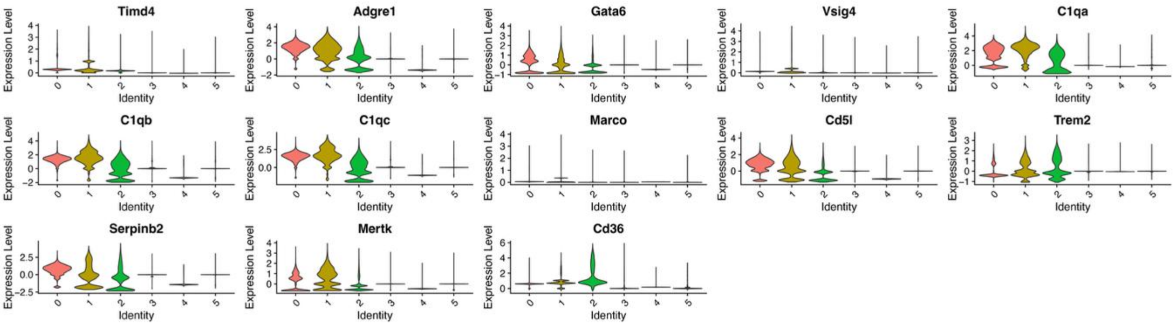

Supplementary Figure 6. Supplementary Figure 6: Resident macrophage markers. Violin plot of genes associated with resident macrophages. Numbers on the x-axis are the cluster number.

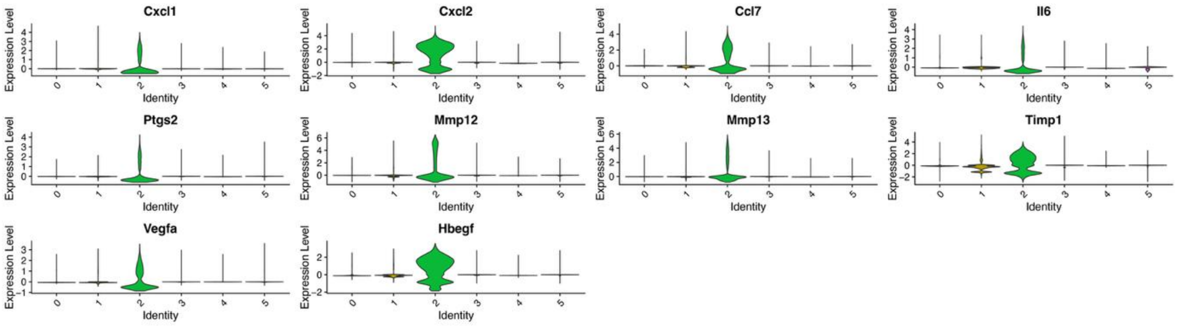

Supplementary Figure 7: Inflammatory macrophage markers. Violin plot of genes associated with inflammatory macrophages. Numbers on the x-axis are the cluster number.

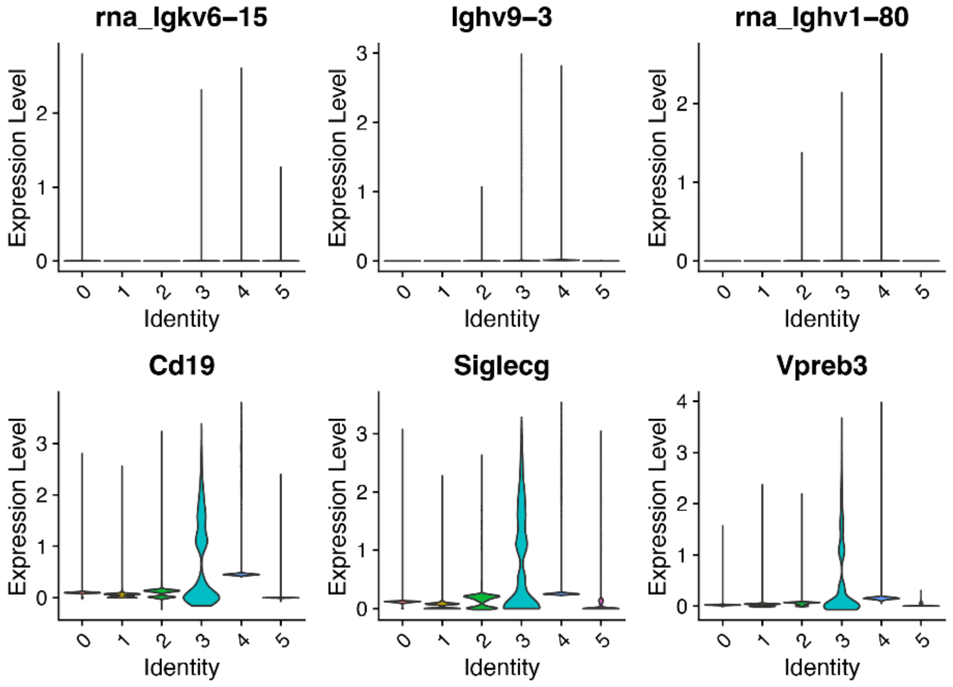

Supplementary Figure 8: Follicular B Cell markers Violin plot of genes associated with Follicular B cells. Numbers on the x-axis are the cluster number.

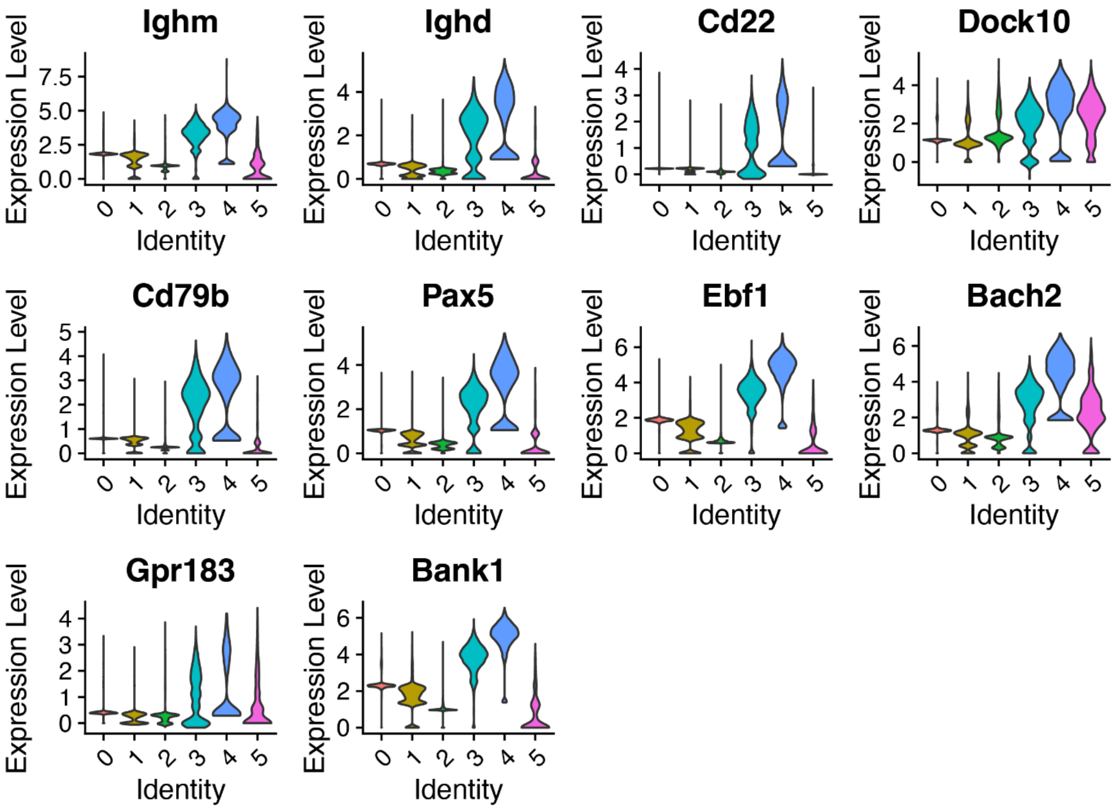

Supplementary Figure 9: Memory B Cell markers. Violin plot of genes associated with Memory B cells. Numbers on the x-axis are the cluster number.

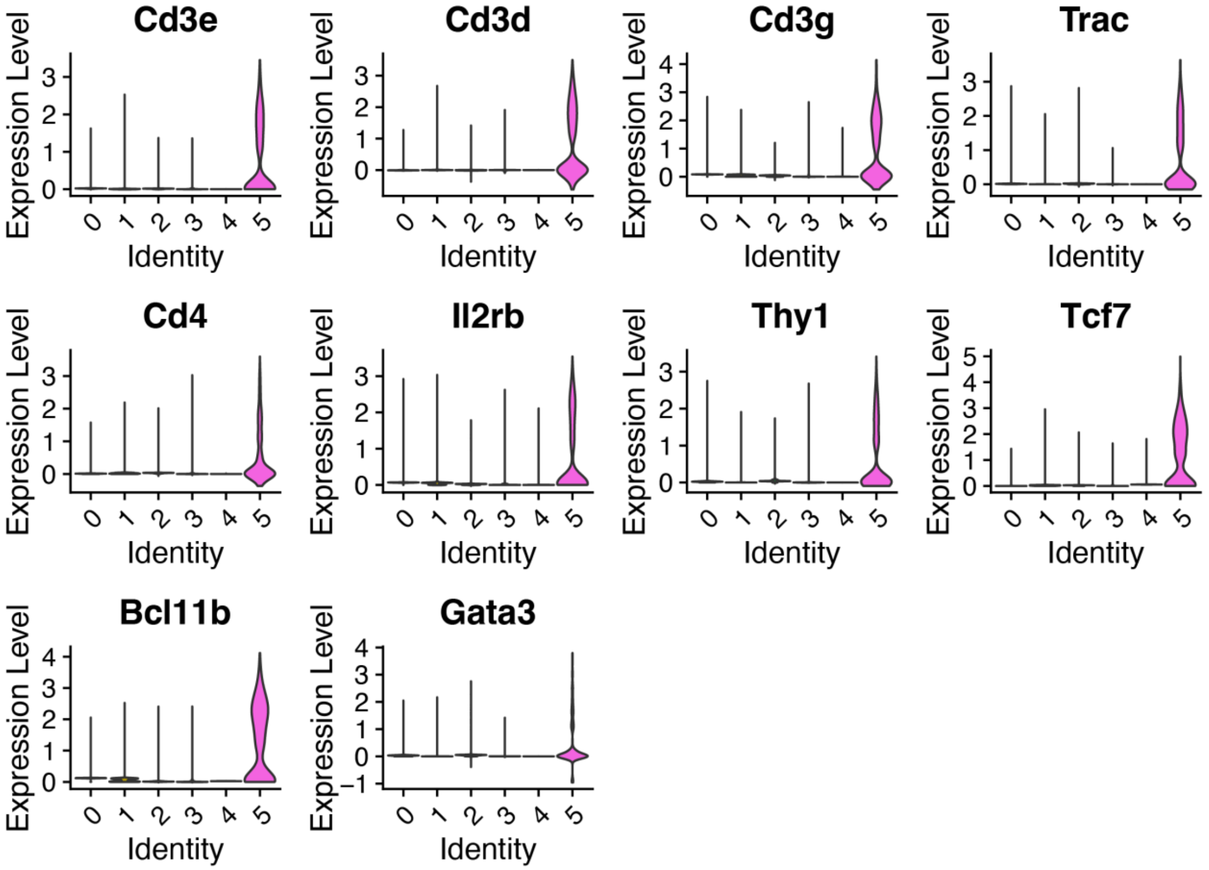

Supplementary Figure 10. CD4 T Cell markers. Violin plot of genes associated with CD4 T cells. Numbers on the x-axis are the cluster number.

Proteomics & additional mAb supplementary figures

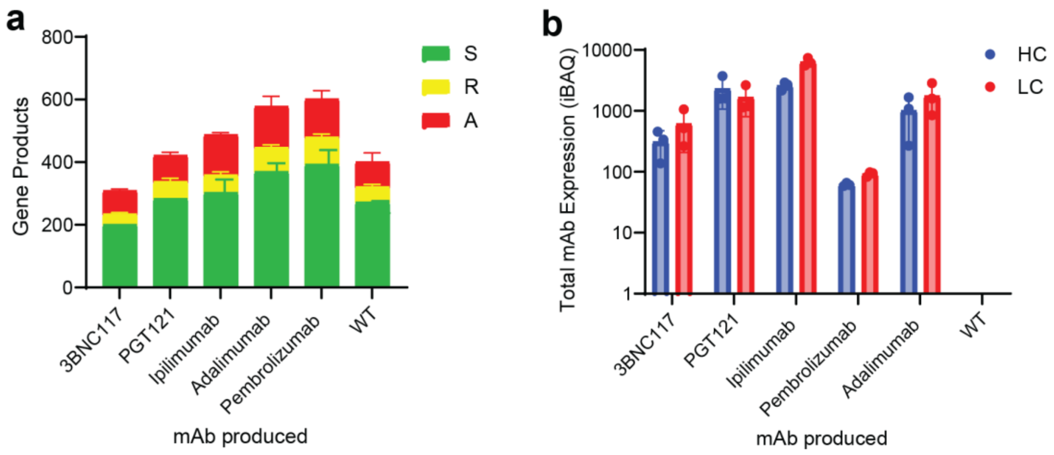

Supplementary Figure 11: Secretomics additional data. a, Identified protein quality across identified gene products **b,** iBAQ scores across mAbs across sample groups.

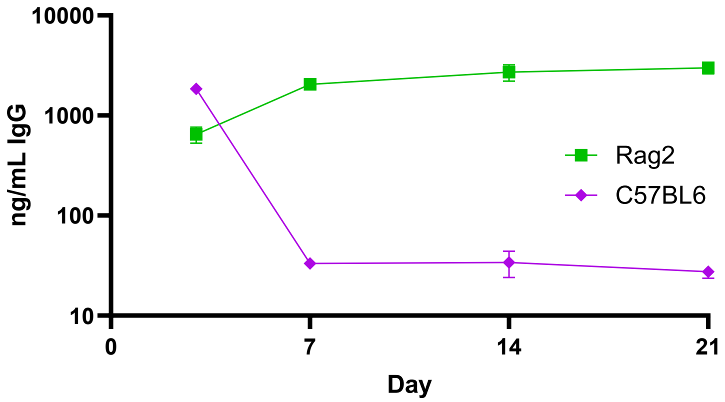

Supplementary Figure 12. Differences in mAb titer and pharmacokinetics in immunocompetent vs immunocompromised mice. Time vs Serum levels of Adalimumab secreting Implants in C57BL6 and RAG2 mice (n=5).

Supplementary H&E Images

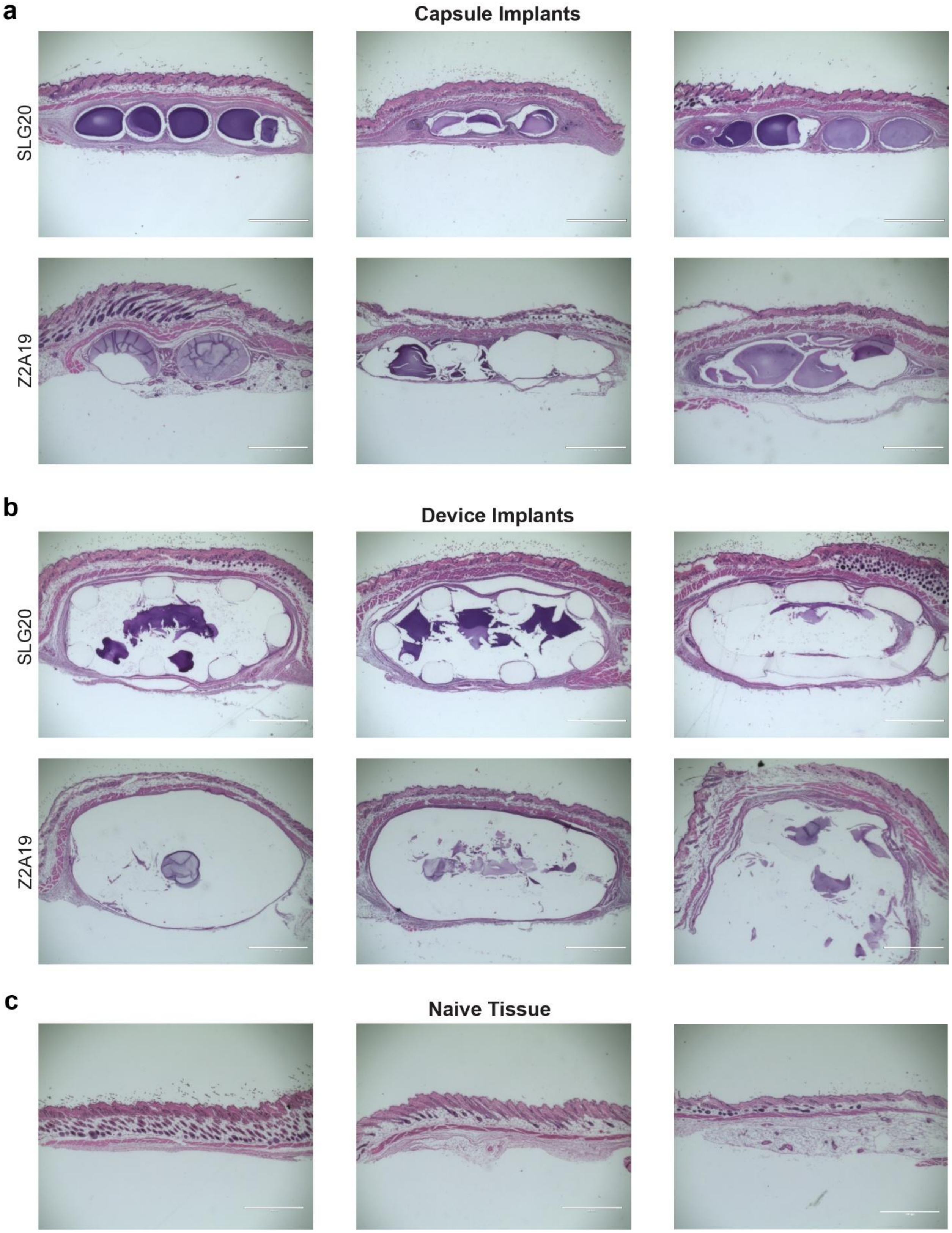

Supplementary Figure 13. Extended H&E images. 4x magnification Images of H&E-stained subcutaneous tissue harvested from C57BL/6 mice implanted with SLG20 or Z2A19 capsules (**A**) or macrodevices (**B**) for 1 month (n=3). Images of Naïve subcutaneous tissue (**C**) are shared for reference. These images were used for fibrotic capsule thickness quantification in figure 4 panels J and K. Scale bars = 1mm.

Supplementary Methods

Small molecule synthesis

All the small molecules were synthesized using click chemistry. The following synthesis route was followed for the synthesis of all the molecules except B2A17. Briefly, to a two necked round bottom flask containing 180 mL of water and methanol in 5:1 ratio under nitrogen was added alkyne followed by addition of tris((1-benzyl-4- triazolyl)methyl) amine (TBTA) (0.25 equiv.), followed by addition of triethylamine (0.25 eq) and copper(I) iodide (0.1 eq) The reaction was then allowed to cool down to 0°C for 15 minutes while still purging the nitrogen. Respective peg- linkers (Azido-PEG-amine) were then added at 1.1 eq to the reaction mixture, and it was allowed to stir for another 5 mins at room temperature followed by heating at 55°C overnight. The reaction was then filtered over celite, solution was evaporated using rotovap and the crude was purified using liquid chromatography with dichloromethane: ultra (22% MeOH in DCM with 3% NH_4OH) 0% to 40% on a 120 gm ISCO silica column.

B2A17 synthesis: 3-iodobenzylamine (1 equiv.) was added to a round bottom flask containing 100 mL of methanol. Triethylamine (2.4 equiv.), sodium azide (2 equiv.), copper iodide (0.15 equiv.), sodium ascorbate (0.1 equiv.), trans-N-N'-dimethylcyclohexene-1,2-diamine (0.2 equiv.) were sequentially added while purging under nitrogen. The reaction was allowed to stir for 10 mins, and 2-ethynyl pyridine (1 equiv.) was added. Reaction was allowed to stir for 5 mins at room temperature followed by overnight stirring at 55°C. Reaction mixture was filtered over Celite, and the solvent was removed using rotovap. The

crude reaction was then purified by liquid chromatography with dichloromethane: ultra (22% MeOH in DCM with 3% NH4OH) 0% to 40% on a 120 gm ISCO silica column.

Z1A34 was synthesized according to the previously reported procedure[1].

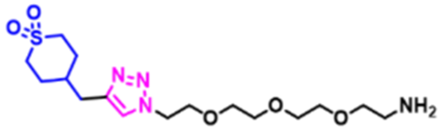

Z1A34

Z2A19 : Corresponding alkyne: 3-ethynyl thiophene, Peg –linker: Azido-PEG2-amine

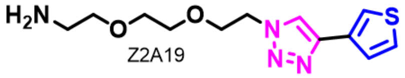

Z2A19

^{1}H NMR (600 MHz; $CDCl_3$): 7.8 (s, 1H, aromatic), 7.6 (d, 1H, aromatic), 7.45 (d, 1H, triazole), 7.36 (m, 1H, aromatic), 4.5 (m, 2H, triazoleCH2), 3.89 (m, 2H, triazole CCH2), 3.62 (m, 2H, NCCH2)), 3.58 (m, 2H, methylene), 3.46 (m, 2H, methylene), 2.83 (m, 2H, NH2CH2), 1.45 (s, 2H, amine)

^{13}C (600 MHz; $CDCl_3$): 143.8(Cq triazole), 131.9 (Cq- thiophene), 126.2 (CH-triazole), 125.8 (Cq-CH, thiophene), 120.9 (Cq-CH-CH, thiophene), 120.7 (CH, thiophene), 72.7 (NCH2C-), 70.5(-OCCH2O-), 70.1(-OCH2-C-O), 69.5 (OC-CH2-Ntriazole), 50.3 (-OCH2C-Ntriazole), 41.5 (NH2-C)

ESI: M+1= 283.1

B2A17: Corresponding alkyne: 2-ethynyl pyridine, Linker: 3-iodobenzyl amine

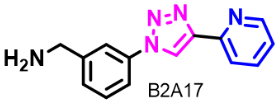

B2A17

^{1}H (600 MHz; $CDCl_3$): 8.6 (d, 2H, aromatic), 8.25 (d, 1H, aromatic), 7.84 (s, 1H, triazole), 7.8 (dd, 1H, aromatic), 7.6 (d, 1H, aromatic), 7.5 (t, 1H, aromatic), 7.4 (d, 1H, aromatic), 7.2 (m, 1H, aromatic), 4.0 (s, 2H, NH2-CH2-Ph), 1.54 (broad s, 2H, NH2).

^{13}C (600 MHz; CDCl3): 150.01(Cq-aromatic), 149.5 (C-N aromatic), 148.9 (Cq-aromatic), 145.56 (CH aromatic), 137.23 (Cq triazole), 136.99 (CH triazole), 129.94 (CH aromatic),

127.54 (CH aromatic), 123.11 (CH aromatic), 120.45 (CH aromatic),120.03(CH aromatic), 119.15 (CH aromatic), 118.76 (CH aromatic), 46.02 (CH2-NH2)

ESI: M+1 = 252.1545

Z1A3 synthesis: Corresponding alkyne: 1-bromo-2-ethynylbenzene, Peg linker: Azido-PEG3-amine

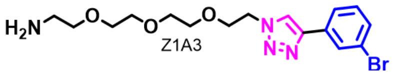

^{1}H (600 MHz; $CDCl_3$): 8.3 (s, 1H, triazole), 8.08 (dd, 1H, aromatic), 7.6 (d, 1H, aromatic), 7.39 (t, 1H, aromatic), 7.18 (t, 1H, aromatic), 4.6 (t, 2H, triazoleCH2), 3.9 (t, 2H, triazole CH2-C), 3.6 (s, 4H, methylene), 3.58 (m, 2H, methylene), 3.56 (m, 2H, methylene), 3.4 (m, 2H,methylene), 2.8 (t, 2H, NH2CH2), 2.08 (s, 2H, amine)

^{13}C (600 MHz; $CDCl_3$): 145.22 (Cq triazole) 133.54 (Cq aromatic), 131.42 (CH aromatic), 130.54 (CH aromatic), 129.19 (Cq triazole), 127.63 (CH aromatic), 124.23 (CH aromatic), 121.13 (CH aromatic), 72.33 (N-CH2-C), 70.61 (O-C-CH2), 70.51(O-C-CH2), 70.43 (O-CH2-C), 70.08 (O-CH2-C), 69.49 (Ntriazole-CH2-C), 50.38 (Ntriazole-CH2), 41.3(NH2CH2)

ESI: M+1= 399.1

Z4A10 : Corresponding alkyne: 1,3 diethynyl benzene, Peg-linker: Azido-PEG4-amine

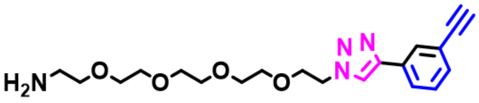

^{1}H (600 MHz; $CDCl_3$): 8.05 (s, 1H, aromatic), 7.9 (s, 1H, triazole), 7.87 (d, 1H, aromatic), 7.44 (d, 1H, aromatic), 7.38 (t, 1H, aromatic), 4.6 (t, 2H, triazoleCH2), 3.9562 (t, 2H, triazoleCH2-C), 3.6289 (d, 8H, methylene), 3.5814 (d, 4H, methylene), 3.49 (t, 2H, methylene), 3.15 (s, 1H, alkyne), 2.85, (t, 2H, NH2CH2), 2.02 (s, 2H, amine)

^{13}C (600 MHz; $CDCl_3$):   146.8 (C triazole), 131.6 (CH aromatic), 131.2 (CH, aromatic), 129.4 (CH aromatic), 128.9 (C triazole), 126.2 (CH aromatic),122.7 (CH aromatic), 121.4 (C, aromatic), 83.4 (C alkyne), 77.6 (CH alkyne), 73.4(NH2-CH2-C), 70.69(O-CH2-C, methylene), 70.67 (O-CH2-C, methylene), 70.66 (OCH2C, methylene) (O-CH2-C,

methylene), 70.63(O-CH2-C), 70.57 (O-C-CH2, methylene), 70.31 (O-C-CH2, methylene), 69.6 (triazole-CH2-C), 50.5 (Ntriazole-CH_2), 41.8 (NH_2-CH_2)

ESI: M+1 = 389.2

Z1A14: Corresponding alkyne: 1-ethynyl-2-fluorobenzene, Peg- linker: Azido-PEG3-amine

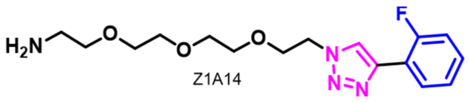

^{1}H (600 MHz ; $CDCl_3$) : 8.3 (t, 1H, aromatic), 8.1 (s, 1H, triazole), 7.2 (m, 2H, aromatic), 7.1 (t, 1H, aromatic), 4.6 (s, 2H, triazole-CH2), 3.9 (s, 2H, triazole-CH2-C), 3.6 (m, 8H, methylene), 3.4 (t, 2H, methylene), 2.8 (s, 2H,NH2-CH2), 2.50 (s, 2H, amine)

^{13}C (600 MHz; $CDCl_3$): 160.01(Cq aromatic), 159.9 (Cq aromatic split), 158.37 (Cq triazole), 158.34 (Cq triazole split), 141.05 (CH triazole), 141.01(CH triazole split), 129.2 (CH aromatic), 127.7(CH aromatic), 124.5 (CH aromatic), 124.0 (CH aromatic), 118.6 (CH aromatic), 115.5 (CH aromatic), 72.25 (NH2-CH2-C), 72.08 (),70.6 (-OC-CH2), 70.5 (OC-CH2), 70.4 (OCH2-C), 70.1 (O-CH2-C), 69.5 (Ntriazole-CH2-C), 50.3 (Ntriazole-CH2), 41.3 (NH2-CH2)

ESI: M+1 = 339.2

Z1A16: Corresponding alkyne: 1-ethynyl-4-fluorobenzene, Peg- linker: Azido-PEG3-amine

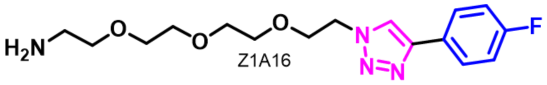

^{1}H (600 MHz ; $CDCl_3$) : 7.99 (s, 1H, triazole), 7.8 (m, 2H, aromatic), 7.1(t, 2H, aromatic), 4.6 (t, 2H, triazole CH2), 3.9 (t, 2H, triazoleCH2C), 3.6 (m, 4H, methylene), 3.5 (m, 4H, methylene), 3.48 (m, 2H, methylene), 2.85 (s, 2H, NH2CH2), 2.42 (s, 2H, amine)

^{13}C (600 MHz; $CDCl_3$): 163.4 (aromatic), 161.78 (aromatic), 146.79 (C-triazole), 127.46 (aromatic), 127.41(aromatic), 127.03 (aromatic), 127.01 (aromatic), 120.85 (CH-triazole), 115.85 (aromatic), 115.7 (aromatic), 71.97 (NH2-CH2-C), 70.49(-O-C-CH2), 70.47 (O-C-

CH2), 70.39 (O-CH2-C), 70.09 (O-CH2-C), 69.49 (Ntriazole-CH2-C), 50.3 (Ntriazole-CH2), 41.2 (NH2-CH2)

ESI: M+1 = 339.2

Z1A17: Corresponding alkyne: 2-ethynyl pyridine, Peg- linker: Azido-PEG3-amine

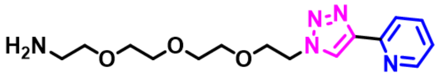

Z1A17

^{1}H (600 MHz ; $CDCl_3$) : 8.56 (d, 1H, aromatic), 8.34 (s, 1H, triazole), 8.08 (d, 1H, aromatic), 7.7 (m, 1H, aromatic), 7.2 (m, 1H, aromatic), 4.6 (m, 2H, triazole CH2), 3.93 (m, 2H,triazoleCH2C), 3.63 (s, 4H, methylene), 3.60 (s, 4H, methylene), 3.50 (m, 2H, methylene), 2.89 (m, 2H, CH2NH2), 2.2 (br s, 2H, amine)

^{13}C (600 MHz; $CDCl_3$): 150.4 (Cq, aromatic), 149.3 (CH, aromatic), 148.2 (CH, aromatic), 136.8 (CH, aromatic), 123.2 (CH, aromatic), 122.7 (CH, aromatic), 120.2(CH, aromatic), 72.7 (NH2CH2-C), 70.6 (-OCCH2), 70.5 (-OCCH2), 70.4 (OCH2C), 70.2 (OCH2C), 69.4 (Ntriazole-CH2-C), 50.4 (Ntriazole-CH2), 41.5 (NH2-CH2)

ESI: M+1 = 322.2

Z4A22: Corresponding alkyne: 4-(prop-2-yn-1-yloxy)aniline, Peg- linker: Azido-PEG4-amine

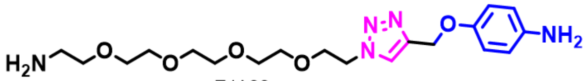

Z4A22

^{1}H (600 MHz ; $CDCl_3$) : 7.79 (s, 1H, triazole), 6.8 (d, 2H, aromatic), 6.62 (d, 2H, aromatic), 5.12 (s, 2H, triazoleCH2), 4.56 (m, 2H, triazoleCH2C), 3.87 (m, 2H, methylene), 3.59 (m, 16H, methylene), 2.91 (br s, 2H, NH2CH2), 2.76 (br s, 2H, amine)

^{13}C (600 MHz; $CDCl_3$): 151.4 (aromatic –OCH), 144.4 (aromatic, triazole –CH), 140.57 (aromatic, NH2-CH), 123.9 (aromatic, triazole-C), 116.3113 (aromatic, NH2-C=C-), 116.07 (aromatic, -OC=C-), 72.6 (-OCH2-triazole), 70.58 (NH2-CH2-C-), 70.5 (-OCH2), 70.4 (-

OCH2-C), 70.18 (-OCH2-C), 69.46 (O-CH2), 62.79 (triazole-CH2-C), 50.3(Ntriazole-CH2), 41.5 (NH2-C).

ESI: M+1 = 410.2

Z4A43: Corresponding alkyne: Ethynylcyclopropane, Peg- linker: Azido-PEG4-amine

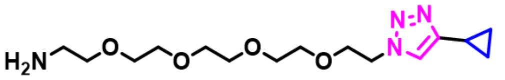

Z4A43

^{1}H (600 MHz ; $CDCl_3$) : 7.4 (s, 1H, triazole), 4.4 (s, 2H, triazoleCH2), 3.76 (m, 2H, triazole CH2C), 3.55 (m, 12H, methylene), 3.47(s, 2H, methylene), 3.16 (s, 2H, methylene), 2.84 (broad s, 2H, amine), 1.85 (m, 1H, cyclic), 0.84 (s, 2H, cyclic), 0.73 (s, 2H, cyclic)

^{13}C (600 MHz; $CDCl_3$): 150.08 (Cq- triazole), 120.9 (CH-triazole), 71.5 (NH2CH2C), 70.5(-OCCH2) 70.47 (-OCCH2), 70.42(OCH2C), 70.09 (OCH2C), 69.6 (triazole-CH2-C), 50.08 (Ntriazole-CH2), 41.1 (NH2-CH2), 7.7 (CH2- cyclic), 6.73 (CH, cyclic).

ESI: M+1 =329.2

Z1A43: Corresponding alkyne: Ethynylcyclopropane, Peg- linker: Azido-PEG3-amine

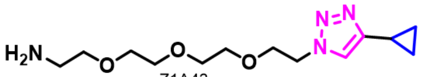

Z1A43

^{1}H (600 MHz ; $CDCl_3$) : 7.4 (s, 1H ,triazole), 4.46 (m, 2H, triazole CH2), 3.8 (m, 2H, triazoleCH2C), 3.58 (s, 8H, methylene), 3.49 (m, 2H, methylene), 2.8 (m, 2H, NH2CH2), 1.9 (m, 1H, cyclic), 1.83 (s, 2H,cmine), 0.9 (m, 2H, cyclic), 0.8 (m, 2H, cyclic)

^{13}C (600 MHz; $CDCl_3$): 149.9(Cq triazole), 120.8(CH triazole), 73.3 (NH2CH2C), 70.5 (-OCCH2), 70.4(-OCH2C), 70.2 (-OCH2C), 69.5 (triazole-CH2-C), 50.0 (Ntriazole-CH2), 41.6 (NH2-CH2), 7.6 (CH2 cyclic), 6.6(CH cyclic)

ESI: M+1 = 285.2

NMR and elemental Analysis of Top Three Molecules

Z2A19 –VLVG

^{1}H (600 MHz, D2O): 8.1 (s, 1H, thiophene), 7.7(s, 1H, thiophene), 7.5 (s, 1H, triazole), 7.4 (s, 1H , thiophene),5-3.9 (m, alginate protons),3.6 (s, 2H, methylene), 3.5 (s, 4H, methylene), 3.5- 3.1 (alginate protons), 2.9 (s, 2H, NH2CH2)

Elemental analysis: %C: 36.7, %H: 4.7, %N:7.4

By elemental analysis there is ~26% modification of the alginate backbone.

Z1A3-VLVG:

^{1}H (600 MHz, D2O): 8.4 (s, 1H, triazole), 7.6 (s, 2H, phenyl), 7.3 (s, 1H, phenyl), 7.2 (s, 1H, phenyl), 4.9- 3.8 (m, alginate protons), 3.6 (m, 8H, methylene) 3.4-3.0 (m, alginate protons), 3.06 (2H, t, NH2CH2)

Elemental: %C: 42, %H: 4.9, %N: 4.5

By elemental analysis there is ~19.2% modification of the alginate backbone.

Z4A10-VLVG:

^{1}H (600 MHz, D2O): 8.1 (s, 1H, triazole), 7.5 (s, 2H, phenyl), 7.3 (s, 2H, phenyl), 5.1-3.82 (m, alginate protons), 3.81 (t, 2H, methylene), 3.4 (m, 8H, methylene), 3.4- 3.1 (m, alginate protons), 2.9 (t, 2H, NH2CH2), 2.7 (s, 1H, alkyne)

Elemental: %C: 39.6, %H: 5.37, %N: 4.9

By elemental analysis, there is ~ 25.8% modification of the alginate backbone.

Device Manufacturing

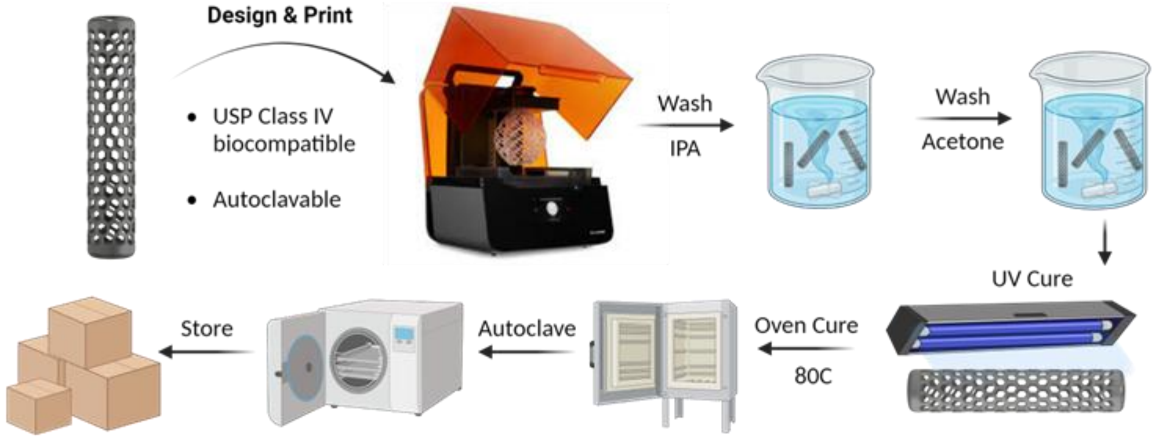

Supplementary Figure 14. Lattice device fabrication and post-print processing method. Lattices are 3D printed from Biomedclear resin using a Formlabs Form 4 resin 3D printer. They then undergo several washing and curing steps before final sterilize.

Reference:

1. Vegas, A. J. *et al.* Combinatorial hydrogel library enables identification of materials that mitigate the foreign body response in primates. *Nat. Biotechnol.* **34**, 345–352 (2016).
